# Endoscopic Endonasal Optic Nerve Decompression for Traumatic Optic Neuropathy: A Systematic Review

**DOI:** 10.22336/rjo.2026.28

**Published:** 2026

**Authors:** Babak Ganjeifar, Seyed Reza Ahmadi Koupaei, Ali Moradi

**Affiliations:** 1 Department of Neurosurgery, Ghaem Hospital, Mashhad University of Medical Sciences, Mashhad, Iran; 2 Department of Emergency Medicine, Faculty of Medicine, Mashhad University of Medical Science, Mashhad, Iran; 3 Clinical Research Development Unit, Ghaem Hospital, Mashhad University of Medical Sciences, Mashhad, Iran; 4 Orthopedic Research Center, Mashhad University of Medical Sciences, Mashhad, Iran

**Keywords:** optic nerve decompression, traumatic optic neuropathy, endoscopic surgery, transsphenoidal surgery, visual recovery, minimally invasive, optic nerve injury, TON = Traumatic optic neuropathy, OND = Optic nerve decompression, RGC = Retinal ganglion cells, RAPD = Relative afferent pupillary defect, VA = Visual acuity, CT = Computed tomography, MRI = Magnetic resonance imaging, EOND = Endoscopic optic nerve decompression

## Abstract

**Introduction:**

Traumatic optic neuropathy (TON) is an acute injury to the optic nerve caused by direct or indirect trauma, often resulting in vision loss. Endoscopic transsphenoidal optic nerve decompression (EOND) is a minimally invasive technique designed to relieve pressure on the optic nerve. This systematic review evaluates the efficacy, safety, and visual outcomes of EOND in patients with TON.

**Methods:**

A systematic search of PubMed, Scopus, and Google Scholar was conducted for articles published between 2014 and 2024. Keywords included “optic nerve decompression”, “traumatic optic neuropathy”, and “endoscopic optic nerve decompression”. After screening 42 articles, 16 studies met the inclusion criteria and were analyzed.

**Results:**

All included studies used the endoscopic transsphenoidal approach. The mean visual recovery rate was 58.4%. All patients received postoperative corticosteroids. Study populations ranged from 11.7 to 68.1 years, including 24 females and 354 males. Postoperative complications were reported in 25% of studies, most commonly cerebrospinal fluid (CSF) leaks.

**Discussion:**

Our findings demonstrated that endoscopic endonasal optic nerve decompression (EOND) is a safe and effective option for traumatic optic neuropathy (TON) patients unresponsive to steroids, with a mean visual recovery rate of 58.4% and a low complication profile, most commonly CSF leaks. While EOND offers clear advantages over traditional approaches, including minimal invasiveness and excellent visualization, the heterogeneity of existing data and the limited number of high-quality studies underscore the need for standardized protocols and further prospective research to optimize patient selection and surgical timing.

**Conclusion:**

EOND is a safe and effective intervention for TON patients unresponsive to medical therapy, offering minimal scarring, improved visualization, and rapid recovery. However, the limited number of studies and the heterogeneity of data highlight the need for further high-quality research to optimize outcomes.

## Introduction

Traumatic optic neuropathy (TON) is a serious complication of craniofacial trauma, occurring in approximately 0.7-2.5% of head injury cases [1]. It involves injury to the optic nerve from direct or indirect trauma to the orbit or skull, often resulting in partial or complete vision loss. Clinical features include ipsilateral vision impairment, color vision changes, pupillary defects, visual field deficits, and, in severe cases, relative afferent pupillary defect (RAPD) [2,3]. Common causes include falls, motor vehicle accidents, and physical assaults [4,5].

TON can be classified as direct or indirect. Direct TON typically results from bony fragment impingement or optic nerve contusion, often causing complete vision loss and a poorer prognosis. Indirect TON preserves anatomical continuity, allowing greater potential for visual recovery [6,7].

No universally accepted treatment protocol exists due to uncertainty regarding optimal timing and approach [8]. Surgical decompression of the optic canal can relieve pressure from hematomas or edema, potentially improving visual outcomes [9,10]. The principles of optic nerve decompression have also been studied in optic nerve compression caused by tumors, showing that relieving mechanical pressure is critical to preserving vision [11].

Three main surgical approaches are described: transorbital (internal/external), transcranial, and endonasal endoscopic transsphenoidal decompression [12]. Cosmetic concerns and increased tissue trauma limit traditional transorbital and transcranial approaches. Advances in endoscopic techniques have made the endonasal approach preferable, providing better visualization, reduced invasiveness, and faster recovery [13,14].

Optical coherence tomography (OCT) has been highlighted in studies published in the *Romanian Journal of Ophthalmology* as a valuable tool for assessing optic nerve structure and function, aiding in diagnosis, monitoring, and prognosis in optic neuropathies [15].

This systematic review evaluates the efficacy, safety, and outcomes of endonasal endoscopic optic nerve decompression for TON, with a focus on visual recovery and complications.

## Methods

We included studies reporting on endoscopic transsphenoidal optic nerve decompression for TON between March 2014 and March 2024. PubMed, Scopus, and Google Scholar were searched without language restrictions.

### Inclusion criteria

- Clinical trials or retrospective analyses of patients undergoing EOND.
- Reported visual recovery and postoperative complications.

### Exclusion criteria

- Studies not using the endoscopic transsphenoidal approach.
- Irrelevant articles.

### Search strategy

**Fig. 1:**
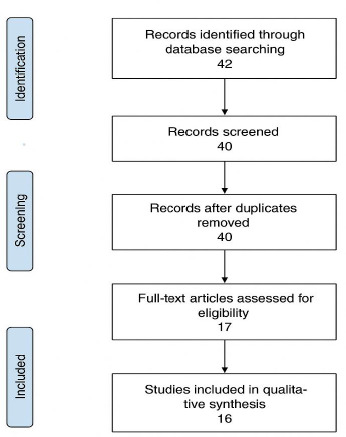
PRISMA flow diagram illustrating the study selection process for the systematic review. A total of 42 records were identified through database searches. After removing duplicates and screening titles/abstracts, 16 studies met the inclusion criteria and were included in the final analysis

Keywords included “optic nerve decompression”, “traumatic optic neuropathy”, and “endoscopic transnasal optic nerve decompression”. Boolean operators refined results. After removing duplicates and screening abstracts, 16 studies were included for full-text review (Fig. 1).

## Results

### Pathophysiology

TON may affect intraocular, intracanalicular, or intracranial segments of the optic nerve [16]. Direct trauma involves foreign body penetration or displaced bone fragments, whereas indirect trauma results from transmitted blunt forces to the orbital apex or optic canal [17-20]. Retinal ganglion cell axons are primarily affected, with over 80% degenerating within one month post-injury [21,22]. Secondary injury arises from edema or vascular compromise [23].

### Diagnosis

Diagnosis relies on ocular trauma history, RAPD, vision loss, color vision changes, and visual field defects [24-28]. RAPD may be the only early sign in mild cases. Neuroimaging (CT or MRI) is recommended for progressive vision loss or surgical planning [29-32].

### Surgical Technique

EOND is performed under general anesthesia. Steps include:

- Accessing the posterior ethmoid region and the sphenoid-ethmoid junction.
- Thinning the medial optic canal wall with a diamond drill.
- Removing bone fragments to decompress the optic nerve.
- Optional intraoperative image guidance enhances precision.

### Outcomes

Analysis of 16 studies showed a mean visual recovery of 58.4%. All patients received postoperative corticosteroids. Patient ages ranged from 11.7 to 68.1 years, with 24 females and 354 males. Postoperative complications occurred in 25% of studies, most commonly CSF leaks (Table 1).

**Table 1:** Summary of the studied article outcomes

Injury to operation duration (day)	Postsurgical recovery rate (%)	Complications	Steroid treatment	Average age	Male/Female	year	Number of patients	study
3-7 days	82/4	NO	yes	26-51	10/2	2014	131	Guy WM et al. [33]
6 hours-96 hours	65	NO	yes	15-76	19/7	2015	26	Emanuelli E et al. [11]
3-7 days	46/9	4 SCF Leak 1 cavernous sinus bleeding	yes	6-62	86/10	2016	96	Yu B et al. [34]
3-7days	45/5	2 SCF Leak	yes	18-67	8/3	2017	11	He Z-H et al. [35]
3-7 days	18/75	NO	yes	50-10	15/1	2017	16	Xie D et al. [36]
4-5 days	78/4	NO	yes	16-50	1004/271	2017	1275	Yan W et al. [37]
3 days	54/84	3 SCF Leak, 1 cavernous sinus bleeding	yes	3-17	53/9	2018	62	Yu B et al. [38]
3 days	80	3 SCF Leak	yes	11-52	11/9	2018	20	Gupta et al. [39]
5-67 days	59/1	NO	yes	1-64	21/1	2020	22	Li J et al. [40]
7-30 days	71/9	Bleeding, 4 SCF Leak, 4 cavernous sinus bleeding	yes	12-28	432/47	2021	479	Zhao S-F et al. [41]
3-7days	54/2	4 SCF Leak	yes	15-73	58/14	2021	72	Yan W et al. [42]
10 days	18/75	NO	yes	14-50	15/1	2021	16	Sun J et al. [43]
2-12 days	85	NO	yes	11-52	16/4	2022	20	Kumar S et al. [44]
-	67/66	5 SCF Leak	yes	7-64	39/5	2022	44	Zhao X et al. [45]
3 days	54/1	NO	yes	19-67	34/3	2022	37	Li X et al. [46]
7-10	59.6	NO	yes	3-12	35/12	2023	47	Li Y et al. [47]

## Discussion

Endoscopic optic nerve decompression (EOND) offers advantages over traditional approaches, including minimal tissue trauma, improved visualization, and shorter operative time. Early intervention is crucial to prevent irreversible optic nerve damage. Image-guided navigation enhances surgical precision and helps avoid injury to critical structures such as the carotid artery [48].

Complications are rare; cerebrospinal fluid (CSF) rhinorrhea is the most common and can be managed promptly. Despite heterogeneous data and limited studies, EOND appears safe and effective for patients with traumatic optic neuropathy (TON) unresponsive to medical therapy.

## Conclusion

The endoscopic transsphenoidal approach provides safe and effective optic nerve decompression, utilizing natural anatomical corridors and minimizing tissue trauma. Adequate knowledge of local anatomy and image-guided navigation reduces risks, particularly regarding the carotid artery.

EOND allows early, effective decompression of the traumatized optic nerve, ensuring exposure of the optic canal and orbital apex with minimal complications. This technique improves visual outcomes in TON patients who do not respond to steroid therapy and represents a valuable surgical option.
